# Association between circulating levels of sex steroid hormones and esophageal adenocarcinoma in the FINBAR Study

**DOI:** 10.1371/journal.pone.0190325

**Published:** 2018-01-17

**Authors:** Jessica L. Petrick, Roni T. Falk, Paula L. Hyland, Patrick Caron, Ruth M. Pfeiffer, Shannon N. Wood, Sanford M. Dawsey, Christian C. Abnet, Philip R. Taylor, Chantal Guillemette, Liam J. Murray, Lesley A. Anderson, Michael B. Cook

**Affiliations:** 1 Division of Cancer Epidemiology and Genetics, National Cancer Institute, NIH, DHHS, Bethesda, Maryland, United States of America; 2 Pharmacogenomics Laboratory, Centre Hospitalier de l’Université Laval de Québec (CHU de Québec) Research Center and Faculty of Pharmacy, Laval University, Québec City, Québec, Canada; 3 Centre for Public Health, School of Medicine, Dentistry and Biomedical Sciences, Queens University Belfast, Belfast, Northern Ireland, United Kingdom; University Hospital Llandough, UNITED KINGDOM

## Abstract

**Background:**

Esophageal adenocarcinoma (EA) is characterized by a strong male predominance. Sex steroid hormones have been hypothesized to underlie this sex disparity, but no population-based study to date has examined this potential association.

**Methods:**

Using mass spectrometry and ELISA, we quantitated sex steroid hormones and sex hormone binding globulin, respectively, in plasma from males– 172 EA cases and 185 controls–within the Factors Influencing the Barrett/Adenocarcinoma Relationship (FINBAR) Study, a case-control investigation conducted in Northern Ireland and Ireland. Multivariable adjusted logistic regression was used to calculate odds ratios (ORs) and 95% confidence intervals (CIs) for associations between circulating hormones and EA.

**Results:**

Higher androgen:estrogen ratio metrics were associated with increased odds of EA (e.g., testosterone:estradiol ratio OR_Q4 v. Q1_ = 2.58, 95%CI = 1.23–5.43; P_trend_ = 0.009). All estrogens and androgens were associated with significant decreased odds of EA. When restricted to individuals with minimal to no decrease in body mass index, the size of association for the androgen:estrogen ratio was not greatly altered.

**Conclusions:**

This first study of sex steroid hormones and EA provides tentative evidence that androgen:estrogen balance may be a factor related to EA. Replication of these findings in prospective studies is needed to enhance confidence in the causality of this effect.

## Introduction

Esophageal adenocarcinoma (EA) is more common in men than women worldwide, with a male-to-female ratio of approximately 4.4 [1]. However, the male-to-female ratio peaks between 50 to 54 years and then decreases [2], suggesting an androgenic effect and perhaps estrogenic protection. Additionally, established risk factors that vary in prevalence by sex, such as smoking and obesity, cannot fully explain the male predominance [3, 4]. Thus, sex steroid hormones have been proposed as a possible explanation of the sex disparity [5]. This hypothesis is supported by sex steroid hormone involvement in the inflammatory process, including associations between testosterone and inflammatory markers [6–9], sex steroid hormone receptor protein expression—specifically estrogen receptor *β*—in esophageal cancer tissue [10, 11], and lower rates of EA among men with prostate cancer, who are likely to receive anti-androgen therapies [12, 13]. Additionally, a small hospital-based study reported higher testosterone levels among EA cases (n = 25) than controls (n = 8) [14], and two studies of the EA precursor metaplasia, Barrett’s esophagus (BE), reported positive associations for free androgens [15, 16]. Thus, we investigated whether circulating sex steroid hormone concentrations were associated with EA in the Factors Influencing the Barrett/Adenocarcinoma Relationship (FINBAR) study–a population-based case-control study of EA and BE conducted in Northern Ireland and the Republic of Ireland between 2002 and 2004 [17–36].

## Materials and methods

### Study population

The aim of the FINBAR study was to determine the stage along the esophageal inflammation-metaplasia-adenocarcinoma sequence that potential risk factors for BE and EA exert their effects [35]. To accomplish this, the study included individuals aged ≤85 years with a histologically confirmed EA (N = 227) and ≥3 cm of visible BE (n = 224). *In situ* cancers were excluded. In Northern Ireland, cases were identified from electronic pathology records from all laboratories in the province. In the Republic of Ireland, cases were identified from primary esophageal cancer treatment centers. The association between sex steroid hormones and BE has previously been reported [16]; therefore, only EA cases and controls are included in the current study.

Eligible controls were adults without a history of gastrointestinal cancer or BE (N = 260). Controls were selected at random from the General Practice Master Index in Northern Ireland and from four general practices in the Republic of Ireland and frequency-matched to cases on sex and 5-year age group.

For the current study of circulating hormones, we restricted the study population to males because there were too few females to provide adequate statistical power for a female-only analysis.

Thus, 172 EA cases and 185 controls are included in the current study. The FINBAR study was approved by the Research Ethics Committee of Queen’s University Belfast, the Clinical Research Ethics Committee of Cork Teaching Hospitals, the Research Ethics Committee Board of St. James’s Hospital, Dublin, and the National Institutes of Health Office of Human Subjects Research.

### Data and sample collection

Information on demographics and risk factors was obtained by a structured computerized interview administered by trained interviewers. Anthropometric variables (i.e., height, weight, waist, and hip circumference) were measured at the time of interview and recalled from 5-years prior to study enrollment. Written informed consent was obtained from each participant before interview.

At the time of interview, a 30 ml sample of peripheral venous blood (non-fasting) was drawn, transported on ice, and then centrifuged within 2 hours for the majority of participants. Serum, plasma, and buffy coat were stored at −80°C. Plasma samples from EDTA-treated tubes were used for quantitation of circulating sex steroid hormones and sex hormone binding globulin (SHBG).

### Laboratory assays and measurements

All assays were performed in 2015 at the Pharmacogenomics Laboratory of Laval University, Quebec, Canada. The plasma samples were never thawed prior to this study and have been stored since processing at −80°C. Prior studies have shown long-term stability of sex steroid hormones at temperatures less than or equal to −20°C [37–43]. Samples were quantitated for dehydroepiandrosterone (DHEA), androstenediol, androstenedione, progesterone, testosterone, dihydrotestosterone (DHT), androsterone (ADT), estrone, and estradiol using gas chromatography–mass spectrometry [44]. In each of the nine batches, three low and three high hormone concentration quality control replicates were included. All hormone coefficients of variation (CVs) were less than 10% (range: 3.5–7.4%).

SHBG was quantitated using ELISA (Diagnostics Biochem Canada, Inc.). In each of the fourteen batches, one low and one high concentration replicate were included and the CV was 8.8%.

In addition to individual hormones, we calculated parent estrogens (the sum of estrone and estradiol), testosterone:parent estrogens ratio, testosterone:estradiol ratio, androstenedione:estrone ratio, free estradiol [45], free testosterone [46], and free DHT [47]. Hormone levels were categorized in quartiles, based on the distributions among the control participants. Quantiles allowed examination of the associations without assumptions about the underlying dose-response relationship. Tests of linear trend were performed based on the quartile-specific medians of the hormone levels.

### Statistical analysis

Unconditional logistic regression was used to calculate odds ratios (ORs) and corresponding 95% confidence intervals (CIs) for the associations between circulating hormones and EA odds. Potential confounders were variables associated with EA and with at least one exposure. These were entered into an age-adjusted multivariable stepwise logistic regression model (entrance p-value = 0.05 and removal p-value = 0.10). Final adjusted models included age (<54.1, 54.1–65.8, 65.9–72.2, ≥72.3), education (<10, 10–12, ≥13 years), smoking status (ever, never), body mass index (BMI, <25.0, 25.0–29.9, ≥30.0 kg/m^2^), frequent gastroesophageal reflux disease (GERD) symptoms (yes, no; defined as heartburn or acid reflux occurring weekly at least 5 years prior to the interview), and serologic *Helicobacter pylori* status (positive, negative) [34]. We additionally tested alcohol consumption, physical activity, occupation, waist-to-hip ratio, aspirin use, proton pump inhibitor use, and H2 receptor antagonist use, but these did not meet the inclusion criteria and were not included in the final models. Additionally, adjustment for age and BMI as continuous instead of categorical measures had negligible effects on the analysis. As androgens and estrogens are known to exhibit circadian variation [48], we also adjusted for blood draw time of day (as minutes and am/pm), and our results were not substantially altered. Effect measure modification of the relationship between hormones–modeled both as continuous and categorical–and EA by age, BMI, smoking, *H*. *pylori*, GERD symptoms, waist-to-hip ratio, proton pump inhibitor use, H2 receptor antagonist use, and geographic location was assessed using likelihood ratio tests [49]. The likelihood ratio tests were corrected for multiple comparisons using a false discovery rate [50]. After correction, there were no effect-measure modifiers of the associations between hormones and EA.

All tests were two-sided. All analyses were conducted using SAS, version 9.3 (SAS Institute, Cary, NC).

### Sensitivity analysis

As the measured hormone levels could be affected by cachexia—or weight loss due to underlying EA—we categorized the cases into three groups: 1) individuals without weight loss between BMI reported 5-years prior to interview and the BMI measured at interview (i.e., BMI change ≥ 0 kg/m^2^); among individuals with weight loss, we dichotomized BMI change at the mean into 2) BMI change <-3.8 kg/m^2^ and 3) BMI change -3.8 to <0 kg/m^2^. This analysis was conducted using polytomous logistic regression. Sample size was limited; thus, we dichotomized the hormone levels. To further examine the potential residual confounding by age, we examined continuous hormone variables, which were standardized to half the value of the interquartile range, and adjusted for continuous age.

## Results

Descriptive characteristics of controls and EA cases are presented in **S1 Table**. Significant differences were noted for GERD symptoms (20% controls v. 48% EA cases), *H*. *pylori* positivity (63% v. 52%), manual occupation (51% v. 63%), ever-smoking (64% v. 84%), and education (11.7 v. 10.6 years). Additionally, significant differences in BMI were noted for both self-reported BMI 5-years prior to enrollment or diagnosis (27.2 v. 28.6 kg/m^2^) and measured at study enrollment (27.9 v. 26.3 kg/m^2^).

As shown in **Table 1**, most of the individual hormones had higher measured concentrations in the controls than in the EA cases. For example, testosterone (12.07 v. 10.83 nmol/L, respectively) and estradiol (67.33 v. 50.54 pmol/L) concentrations were both higher in controls than cases. However, the androgen:estrogen ratio metrics were lower in controls compared to cases (e.g., testosterone:estradiol ratio 177.92 v. 209.59).

**Table 1:** Mean circulating metabolite concentrations among esophageal adenocarcinoma cases and controls, in the FINBAR Study: 2002–2004.

	Controls			Esophageal Adenocarcinoma Cases			*P-value*^a^
Hormone	N	Median	Interquartile Range	N	Median	Interquartile Range	
DHEA, nmol/L	185	5.13	3.36–8.18	169	3.68	1.87–5.55	<0.0001
Androstenediol, pmol/L	183	1764.39	1315.76–2494.35	164	1286.03	848.78–1913.04	<0.0001
Androstenedione, nmol/L	185	2.72	2.03–3.39	171	2.34	1.68–3.28	0.06
Testosterone, nmol/L	184	12.07	9.67–15.20	170	10.83	7.52–14.77	0.01
DHT, pmol/L	184	1008.55	751.13–1372.98	171	746.69	548.68–1193.90	<0.0001
ADT, pmol/L	174	592.55	409.10–754.82	143	466.67	315.11–643.47	<0.0001
Estrone, pmol/L	185	104.23	81.11–129.60	165	89.99	65.21–126.75	0.0009
Estradiol, pmol/L	185	67.33	55.29–82.60	170	50.54	37.48–66.49	<0.0001
Progesterone, nmol/L	90	0.23	0.19–0.36	91	0.26	0.20–0.34	0.3
SHBG, nmol/L	184	53.70	39.50–69.35	172	71.10	48.65–100.90	<0.0001
Parent estrogens, pmol/L	185	175.22	142.98–210.75	165	139.37	106.03–196.09	<0.0001
Testosterone: Parent estrogens ratio	184	68.78	54.55–87.32	165	78.88	52.38–102.22	0.05
Androstenedione: Estrone ratio	185	24.49	20.59–31.03	165	26.16	18.98–37.99	0.2
Testosterone: Estradiol ratio	184	177.92	140.11–221.56	170	209.59	157.47–279.48	0.0002
Free testosterone, nmol/L	183	0.18	0.14–0.22	170	0.13	0.09–0.17	<0.0001
Free DHT, pmol/L	183	16.35	11.99–20.41	170	10.21	6.50–13.93	<0.0001
Free estradiol, pmol/L	184	1.51	1.27–1.85	170	0.99	0.73–1.42	<0.0001

^a^Wilcoxon-Mann-Whitney test comparing the medians of cases and controls.

Individual sex steroid hormones, and calculated free estradiol, free testosterone, and free DHT, were associated with significantly decreased EA odds (**Table 2**). However, SHBG and the androgen:estrogen ratio metrics–particularly testosterone:estradiol–were associated with an increased EA odds. Comparing quartile 4 versus 1, SHBG was associated with 2.3-times the odds of EA (OR = 2.30, 95% CI = 1.10–4.80; P_trend_ = 0.01), and the testosterone:estradiol ratio was associated with 2.6-times the odds (OR = 2.58, 95% CI = 1.23–5.43; P_trend_ = 0.009). Adjustment for covariates had little effect on the estimates, as observed in the unadjusted results (**S2 Table**).

**Table 2:** Adjusted^a^ odds ratios (ORs) and 95% confidence intervals (CIs) for the associations between circulating metabolite concentrations and esophageal adenocarcinoma incidence, in the FINBAR Study: 2002–2004.

Hormone	Control (n)	Esophageal Adeno. (n)	OR	95% CI
**DHEA, nmol/L**				
<3.36	45	70	Referent	
3.36 to <5.13	38	40	0.91	(0.46, 1.80)
5.13 to <8.18	46	29	0.43	(0.21, 0.90)
≥8.18	43	16	0.32	(0.14, 0.76)
*P trend*^*b*^				0.003
**Androstenediol, pmol/L**				
<1315.76	41	79	Referent	
1315.76 to <1764.39	42	34	0.41	(0.21, 0.80)
1764.39 to <2494.35	44	27	0.33	(0.16, 0.68)
≥2494.35	43	12	0.13	(0.05, 0.30)
*P trend*^*b*^				<0.0001
**Androstenedione, nmol/L**				
<2.03	45	57	Referent	
2.03 to <2.72	42	39	0.96	(0.48, 1.92)
2.72 to <3.39	41	26	0.57	(0.27, 1.19)
≥3.39	44	35	0.65	(0.32, 1.31)
*P trend*^*b*^				0.1
**Testosterone, nmol/L**				
<9.67	42	66	Referent	
9.67 to <12.07	45	28	0.54	(0.27, 1.07)
12.07 to <15.20	42	30	0.45	(0.22, 0.92)
≥15.20	42	32	0.50	(0.25, 1.01)
*P trend*^*b*^				0.05
**DHT, pmol/L**				
<751.13	42	80	Referent	
751.13 to <1008.55	43	29	0.39	(0.19, 0.78)
1008.55 to <1372.98	42	21	0.23	(0.11, 0.50)
≥1372.98	44	27	0.23	(0.11, 0.49)
*P trend*^*b*^				<0.0001
**ADT, pmol/L**				
<409.10	38	51	Referent	
409.10 to <592.55	43	44	0.73	(0.37, 1.44)
592.55 to <754.82	42	21	0.40	(0.19, 0.87)
≥754.82	41	15	0.28	(0.12, 0.64)
*P trend*^*b*^				0.0008
**Estrone, pmol/L**				
<81.11	41	72	Referent	
81.11 to <104.23	45	31	0.34	(0.17, 0.68)
104.23 to <129.60	42	19	0.24	(0.11, 0.52)
≥129.60	44	29	0.36	(0.18, 0.72)
*P trend*^*b*^				0.003
**Estradiol, pmol/L**				
<55.29	46	97	Referent	
55.29 to <67.33	42	21	0.25	(0.12, 0.52)
67.33 to <82.60	41	16	0.20	(0.09, 0.44)
≥82.60	43	22	0.25	(0.12, 0.51)
*P trend*^*b*^				<0.0001
**Progesterone, nmol/L**^**c**^				
<0.15	86	74	Referent	
0.15 to <0.20	29	21	0.74	(0.35, 1.54)
0.20 to <0.29	29	33	1.58	(0.80, 3.14)
≥0.29	28	30	1.00	(0.49, 2.01)
*P trend*^*d*^				0.9
**SHBG, nmol/L**				
<39.50	43	27	Referent	
39.50 to <53.70	42	28	0.97	(0.44, 2.13)
53.70 to <69.35	44	23	0.64	(0.28, 1.44)
≥69.35	42	80	2.30	(1.10, 4.80)
*P trend*^*b*^				0.01
**Parent estrogens, pmol/L**				
<142.98	43	85	Referent	
142.98 to <175.22	43	23	0.29	(0.14, 0.61)
175.22 to <210.75	42	19	0.22	(0.10, 0.47)
≥210.75	44	24	0.26	(0.13, 0.54)
*P trend*^*b*^				<0.0001
**Testosterone: Parent estrogens ratio**				
<54.55	45	35	Referent	
54.55 to <68.78	39	23	0.91	(0.42, 2.00)
68.78 to <87.32	44	31	0.93	(0.43, 1.99)
≥87.32	43	62	2.06	(1.00, 4.24)
*P trend*^*b*^				0.03
**Androstenedione: Estrone ratio**				
<20.59	41	41	Referent	
20.59 to <24.49	43	18	0.54	(0.24, 1.20)
24.49 to <31.03	44	31	0.92	(0.44, 1.92)
≥31.03	44	61	1.56	(0.76, 3.22)
*P trend*^*b*^				0.1
**Testosterone: Estradiol ratio**				
<140.11	44	28	Referent	
140.11 to <177.92	40	28	1.31	(0.59, 2.91)
177.92 to <221.56	44	26	1.10	(0.49, 2.48)
≥221.56	43	74	2.58	(1.23, 5.43)
*P trend*^*b*^				0.009
**Free testosterone, nmol/L**				
<0.14	41	91	Referent	
0.14 to <0.18	45	28	0.31	(0.16, 0.61)
0.18 to <0.22	42	24	0.26	(0.12, 0.53)
≥0.22	42	13	0.16	(0.07, 0.37)
*P trend*^*b*^				<0.0001
**Free DHT, pmol/L**				
<11.99	42	101	Referent	
11.99 to <16.35	41	32	0.24	(0.12, 0.49)
16.35 to <20.41	44	12	0.09	(0.04, 0.21)
≥20.41	43	11	0.07	(0.03, 0.18)
*P trend*^*b*^				<0.0001
**Free estradiol, pmol/L**				
<1.27	44	106	Referent	
1.27 to <1.51	41	21	0.24	(0.12, 0.50)
1.51 to <1.85	44	16	0.20	(0.09, 0.42)
≥1.85	42	13	0.15	(0.07, 0.34)
*P trend*^*b*^				<0.0001

^a^Logistic regression models were adjusted for age at interview (quartiles), education (<10, 10–12, 13–20 years), smoking (ever/never), BMI at interview (<25, 25–<30, ≥30 kg/m^2^), gastroesophageal reflux disease symptoms (yes/no), and *H*. *pylori* seropositivity (yes/no).

^b^Tests of linear trend were calculated by assigning the median of each quartile as scores.

^c^Progesterone values below the LOD form the referent with the subsequent three categories based on tertiles of the observed population distribution.

^d^Test of linear trend for progesterone was calculated by assigning the categorical groups as scores.

When we examined the cases by BMI change, the androgen:estrogen ratio metrics were still associated with an increased odds of EA among individuals with little to no BMI change during the 5 years preceding interview. Comparing individuals with ≥ median versus < median, testosterone:estradiol ratio was associated with 1.4 to 2.6-times the odds of EA among individuals with little to no BMI change (OR = 1.35, 95% CI = 0.54–3.39; P_trend_ = 0.3 among EA cases with a BMI change ≥0 kg/m^2^ and OR = 2.61, 95% CI = 1.27–5.38; P_trend_ = 0.0005 among individuals with a BMI change of -3.8 to <0 kg/m^2^) (**S3 Table**). Additional adjustment of the continuous hormones and SHBG for continuous age did not substantially alter the results (**S4 Table**).

## Discussion

This is the first population-based study to examine associations between circulating sex steroid hormones and EA and provides tentative evidence that androgen:estrogen balance may be a factor related to EA. In our analysis, a high ratio of androgens to estrogens–particularly testosterone:estradiol ratio–was more common in EA patients than controls, including after restriction to cases without weight loss in the previous 5 years. The highest quartile ratio of testosterone:estradiol was associated with a 2.4-times increased odds of EA, while androstenedione:estrone and testosterone:parent estrogens ratios were associated with a non-significant 1.6-times odds. However, many of the individual hormone metrics assessed were inversely associated with EA. Deciphering the extent to which these associations are due to reverse causation will require prospective studies with prediagnostic hormone quantitation.

In older men, obesity is strongly linked to SHBG and influences levels of circulating sex hormones. Specifically, higher BMI is associated with lower levels of DHEA, testosterone, and SHBG and higher levels of estrogens [51]. In our main analysis, only 23% of the cases did not experience weight loss during the 5-years prior to cancer diagnosis. Cancer patients often present with cachexia, which is associated with hypogonadism. Thus, we were concerned that our findings could be a consequence of the cancer itself. Indeed, studies suggest testosterone levels are lower and SHBG higher in male cancer cases compared to age-matched healthy men [52–54]. As a sensitivity analysis, we examined cases by BMI changes in the 5-years prior to study entry. The results showed that SHBG was associated with increased odds of EA among individuals with the most extreme BMI loss (i.e., <-3.8 kg/m^2^), but not among individuals with little to no BMI loss. Among individuals with little to no BMI change, the ratios of androgens:estrogens were associated with increased odds of EA, but not among individuals with extreme BMI loss. However, cautious interpretation is warranted, as these were *post hoc* analyses that used a proxy (i.e., BMI) of cancer’s metabolic effects.

While it has long been hypothesized that sex steroid hormones may underlie the sex disparities in EA pathogenesis [5], no population-based study to date has examined circulating sex steroid hormones and EA. One small hospital-based study (n = 25 cases) that examined the association between fasting serum testosterone reported that EA cases had significantly higher testosterone concentrations compared with eight control subjects. However, post-esophagectomy testosterone levels in cases decreased to levels similar to controls, leading the authors to conclude that the cancer was causing higher circulating testosterone concentrations [14]. While stage information and treatment status was not uniformly collected in the current study, blood samples were collected prior to treatment for the majority of participants. Therefore, changes in testosterone levels post-treatment are unlikely to completely explain the inverse association between testosterone and EA in our main analysis.

Additionally, BE has been examined in relation to circulating sex steroid hormones in two studies–one from a U.S. military medical center and one from the FINBAR Study. Both studies determined that free androgens were associated with increased odds of BE, although this was limited to participants with a high waist-to-hip ratio in the FINBAR Study [15, 16]. Thus, free androgens may be important in the development of BE–possibly by delaying wound healing and allowing more time for metaplasia to develop–while the balance of androgens and estrogens is tentatively inferred from this study to be a factor for progression to invasive cancer.

*In vitro* studies of esophageal squamous cell carcinoma [55] and adenocarcinoma [56] have shown that administration of estradiol decreases cell growth. One of these studies further examined the effects of estradiol and DHT administration on EA tumors in mice and reported that estradiol injections resulted in a high estradiol:DHT ratio and significant inhibition of tumor growth [55]. This indirectly further supports that the androgen:estrogen balance may be important for EA development, whereby higher levels of androgens relative to estrogens may favor cellular proliferation and tumor growth.

A potential limitation of the current study is that the plasma for cases was collected at time of interview. While the esophagus is not an endocrine gland, there is the possibility that circulating hormone concentrations could be affected by tumor growth and reverse causation could be contributing to these findings [14]. Additionally, age and chronic disease status may alter hormone and SHBG levels. We adjusted the models for age and GERD, which has been suggested to be associated with altered hormone levels [57], but there still may be residual confounding. There is also a potential in this study for recall bias, or differential recall between cases and controls, particularly for weight 5 years prior to study interview. A strength of the current study is that the FINBAR Study was designed specifically to examine risk factors for EA–such as GERD and *H*. *pylori*, and we were able to adjust for these. The current study also used robust quantitative technology for sex steroid hormone measurement.

In summary, this study provides tentative evidence that the balance of androgens to estrogens may be important in the development of EA. However, the large number of inverse associations detected make the possibility of reverse causation more likely and underscore the need for a cautious interpretation. Future studies need to be conducted using prospectively collected blood to provide additional confidence that these associations between sex steroid hormones and EA represent causal effects and not effects of cachexia.

## Supporting information

S1 Table Distribution of characteristics among control participants and esophageal adenocarcinoma cases, in the FINBAR Study2002–2004. ^a^P values based on t-test for continuous variables and χ^2^ test for categorical variables. ^b^Defined as at least 50 times per year or about once a week. ^c^Defined as at least once weekly for 6 months or more.
(DOCX)

S2 Table Unadjusted odds ratios (ORs) and 95% confidence intervals (CIs) for the associations between circulating metabolite concentrations and esophageal adenocarcinoma incidence, in the FINBAR Study: 2002–2004. ^a^Tests of linear trend were calculated by assigning the median of each quartile as scores. ^b^Progesterone values below the LOD form the referent with the subsequent three categories based on tertiles of the observed population distribution. ^c^Test of linear trend for progesterone was calculated by assigning the categorical groups as scores.
(DOCX)

S3 Table Adjusted^a^ odds ratios (ORs) and 95% confidence intervals (CI) for associations between circulating sex steroid hormone concentrations and esophageal adenocarcinoma risk, stratified by body mass index (kg/m^2^) change in the 5 years preceding interview. ^a^Logistic regression models were adjusted for age at interview (quartiles), education (<10, 10–12, 13–20 years), smoking (ever/never), BMI at interview (<25, 25–<30, ≥30 kg/m2), gastroesophageal reflux disease symptoms (yes/no), and *H*. *pylori* seropositivity (yes/no). ^b^Tests of linear trend were calculated by assigning the median of each quartile as scores. ^c^Progesterone values below the LOD form the referent with the subsequent three categories based on tertiles of the observed population distribution. ^d^Test of linear trend for progesterone was calculated by assigning the categorical groups as scores.
(DOCX)

S4 Table Adjusted^a^ odds ratios (ORs) and 95% confidence intervals (CIs) for the associations between circulating metabolite concentrations^b^ and esophageal adenocarcinoma incidence, Factors Influencing the Barrett/Adenocarcinoma Relationship: 2002–2004. ^a^Logistic regression models were adjusted for age at interview (continuous), education (<10, 10–12, 13–20 years), smoking (ever/never), BMI at interview (<25, 25–<30, ≥30 kg/m2), gastroesophageal reflux disease symptoms (yes/no), and *H*. *pylori* seropositivity (yes/no). ^b^Standardized to half the value of the interquartile range (e.g., for DHEA the OR is 0.70 for an increase of 2.41 nmol/L, which is 0.5*[8.18−3.36]), which approximates a single quartile increase in exposure.
(DOCX)

## Abbreviations

ADT: androsterone
BE: Barrett’s esophagus
CV: coefficient of variation
DHEA: dehydroepiandrosterone
DHT: dihydrotestosterone
EA: esophageal adenocarcinoma
FINBAR: Factors Influencing the Barrett/Adenocarcinoma Relationship
GERD: gastroesophageal reflux disease
SHBG: sex hormone binding globulin
