# Supplementary material for: Association between circulating levels of sex steroid hormones and esophageal adenocarcinoma in the FINBAR Study
Source: PLoS One. 2018 Jan 17;13(1):e0190325. doi: 10.1371/journal.pone.0190325 (PMC5771564; doi:10.1371/journal.pone.0190325)
Supplement: S1 Table — aP values based on t-test for continuous variables and χ2 test for categorical variables. bDefined as at least 50 times per year or about once a week. cDefined as at least once weekly for 6 months or more. (DOCX) [file pone.0190325.s001.docx]

| **S1 Table.** Distribution of characteristics among control participants and esophageal adenocarcinoma cases, in the FINBAR Study2002–2004. | | | |
| --- | --- | --- | --- |
| **Variable** | **Control (n=185)** | **Esophageal  Adenocarcinoma (n=172)** | **P value^a^** |
| Mean age at interview, *y* (SD) | 63.5 (12.6) | 64.3 (10.9) | 0.5 |
| Body mass index, *kg/m^2^* (SD) |  |  |  |
| Measured at study enrollment | 27.9 (4.0) | 26.3 (4.9) | 0.0006 |
| Five-years prior to study  enrollment | 27.2 (4.0) | 28.6 (4.6) | 0.003 |
| Waist-to-hip ratio (SD) | 0.97 (0.07) | 1.02 (0.73) | 0.3 |
| Ever-smoked, *%* | 64.4 | 83.9 | <0.0001 |
| Alcohol use, *g/day* (SD) | 20.6 (23.7) | 16.5 (21.4) | 0.09 |
| Experienced GERD symptoms, *%* | 20.0 | 48.3 | <0.0001 |
| Frequently experienced heartburn, *%* | 18.9 | 45.9 | <0.0001 |
| Often experienced reflux, *%* | 14.1 | 31.6 | <0.0001 |
| Aspirin regular use, *%*^b^ | 31.0 | 28.1 | 0.5 |
| *H pylori* seropositivity, *%* | 63.0 | 52.4 | 0.04 |
| Education, *y* (SD) | 11.7 (3.1) | 10.6 (2.7) | 0.0006 |
| Manual occupation, *%* | 50.6 | 62.6 | 0.03 |
| Physical Activity, *%* |  |  |  |
| Not at all active | 6.0 | 4.9 |  |
| Not very active | 31.0 | 35.8 |  |
| Fairly active | 63.1 | 59.3 | 0.6 |

^a^P values based on t-test for continuous variables and χ^2^ test for categorical variables. ^b^Defined as at least 50 times per year or about once a week. ^c^Defined as at least once weekly for 6 months or more.
