# Supplementary material for: Association between circulating levels of sex steroid hormones and esophageal adenocarcinoma in the FINBAR Study
Source: PLoS One. 2018 Jan 17;13(1):e0190325. doi: 10.1371/journal.pone.0190325 (PMC5771564; doi:10.1371/journal.pone.0190325)
Supplement: S2 Table — aTests of linear trend were calculated by assigning the median of each quartile as scores. bProgesterone values below the LOD form the referent with the subsequent three categories based on tertiles of the observed population distribution. cTest of linear trend for progesterone was calculated by assigning the categorical groups as scores. (DOCX) [file pone.0190325.s002.docx]

**S2** **Table.** Unadjusted odds ratios (ORs) and 95% confidence intervals (CIs) for the associations between circulating metabolite concentrations and esophageal adenocarcinoma incidence, in the FINBAR Study: 2002–2004.

| **Hormone** | **Control (n)** | **Esophageal**  **Adeno. (n)** | **OR** | **95% CI** |
| --- | --- | --- | --- | --- |
| **DHEA, nmol/L** |  |  |  |  |
| <3.36 | 50 | 77 | Referent |  |
| 3.36 to <5.13 | 40 | 42 | 0.68 | (0.39, 1.19) |
| 5.13 to <8.18 | 49 | 34 | 0.45 | (0.26, 0.79) |
| ≥8.18 | 46 | 16 | 0.23 | (0.12, 0.44) |
| *P trend^b^* |  |  |  | <0.0001 |
| **Androstenediol, pmol/L** |  |  |  |  |
| <1315.76 | 45 | 84 | Referent |  |
| 1315.76 to <1764.39 | 46 | 36 | 0.42 | (0.24, 0.74) |
| 1764.39 to <2494.35 | 46 | 30 | 0.35 | (0.19, 0.63) |
| ≥2494.35 | 46 | 14 | 0.16 | (0.08, 0.33) |
| *P trend^b^* |  |  |  | <0.0001 |
| **Androstenedione, nmol/L** | |  |  |  |
| <2.03 | 48 | 63 | Referent |  |
| 2.03 to <2.72 | 46 | 43 | 0.71 | (0.41, 1.25) |
| 2.72 to <3.39 | 46 | 27 | 0.45 | (0.24, 0.82) |
| ≥3.39 | 45 | 38 | 0.64 | (0.36, 1.14) |
| *P trend^b^* |  |  |  | 0.04 |
| **Testosterone, nmol/L** |  |  |  |  |
| <9.67 | 46 | 72 | Referent |  |
| 9.67 to <12.07 | 48 | 29 | 0.39 | (0.21, 0.70) |
| 12.07 to <15.20 | 44 | 34 | 0.49 | (0.28, 0.88) |
| ≥15.20 | 46 | 35 | 0.49 | (0.27, 0.86) |
| *P trend^b^* |  |  |  | 0.04 |
| **DHT, pmol/L** |  |  |  |  |
| <751.13 | 46 | 87 | Referent |  |
| 751.13 to <1008.55 | 46 | 32 | 0.37 | (0.21, 0.65) |
| 1008.55 to <1372.98 | 46 | 21 | 0.24 | (0.13, 0.45) |
| ≥1372.98 | 46 | 31 | 0.36 | (0.20, 0.64) |
| *P trend^b^* |  |  |  | <0.0001 |
| **ADT, pmol/L** |  |  |  |  |
| <409.10 | 43 | 55 | Referent |  |
| 409.10 to <592.55 | 44 | 48 | 0.85 | (0.48, 1.51) |
| 592.55 to <754.82 | 43 | 22 | 0.40 | (0.21, 0.77) |
| ≥754.82 | 44 | 18 | 0.32 | (0.16, 0.63) |
| *P trend^b^* |  |  |  | <0.0001 |
| **Estrone, pmol/L** |  |  |  |  |
| <81.11 | 45 | 74 | Referent |  |
| 81.11 to <104.23 | 48 | 32 | 0.41 | (0.23, 0.72) |
| 104.23 to <129.60 | 45 | 20 | 0.27 | (0.14, 0.51) |
| ≥129.60 | 47 | 39 | 0.50 | (0.29, 0.89) |
| *P trend^b^* |  |  |  | 0.01 |
| **Estradiol, pmol/L** |  |  |  |  |
| <55.29 | 48 | 104 | Referent |  |
| 55.29 to <67.33 | 45 | 25 | 0.26 | (0.14, 0.47) |
| 67.33 to <82.60 | 45 | 16 | 0.16 | (0.08, 0.32) |
| ≥82.60 | 47 | 25 | 0.25 | (0.14, 0.44) |
| *P trend^b^* |  |  |  | <0.0001 |
| **Progesterone, nmol/L^c^** |  |  |  |  |
| <0.15 | 95 | 81 | Referent |  |
| 0.15 to <0.20 | 30 | 21 | 0.82 | (0.44, 1.54) |
| 0.20 to <0.29 | 30 | 36 | 1.41 | (0.80, 2.48) |
| ≥0.29 | 30 | 34 | 1.33 | (0.75, 2.36) |
| *P trend^d^* |  |  |  | 0.2 |
| **SHBG, nmol/L** |  |  |  |  |
| <39.50 | 46 | 27 | Referent |  |
| 39.50 to <53.70 | 46 | 30 | 1.11 | (0.57, 2.15) |
| 53.70 to <69.35 | 46 | 24 | 0.89 | (0.45, 1.76) |
| ≥69.35 | 46 | 91 | 3.37 | (1.86, 6.10) |
| *P trend^b^* |  |  |  | <0.0001 |
| **Parent estrogens, pmol/L** | |  |  |  |
| <142.98 | 46 | 87 | Referent |  |
| 142.98 to <175.22 | 46 | 25 | 0.29 | (0.16, 0.53) |
| 175.22 to <210.75 | 46 | 21 | 0.24 | (0.13, 0.45) |
| ≥210.75 | 47 | 32 | 0.36 | (0.20, 0.64) |
| *P trend^b^* |  |  |  | <0.0001 |
| **Testosterone: Parent estrogens ratio** | | |  |  |
| <54.55 | 46 | 43 | Referent |  |
| 54.55 to <68.78 | 46 | 23 | 0.53 | (0.28, 1.03) |
| 68.78 to <87.32 | 46 | 34 | 0.79 | (0.43, 1.45) |
| ≥87.32 | 46 | 65 | 1.51 | (0.86, 2.65) |
| *P trend^b^* |  |  |  | 0.03 |
| **Androstenedione: Estrone ratio** | |  |  |  |
| <20.59 | 46 | 52 | Referent |  |
| 20.59 to <24.49 | 46 | 19 | 0.37 | (0.19, 0.71) |
| 24.49 to <31.03 | 46 | 33 | 0.63 | (0.35, 1.15) |
| ≥31.03 | 47 | 61 | 1.15 | (0.66, 1.99) |
| *P trend^b^* |  |  |  | 0.3 |
| **Testosterone: Estradiol ratio** | |  |  |  |
| <140.11 | 46 | 32 | Referent |  |
| 140.11 to <177.92 | 46 | 30 | 0.94 | (0.49, 1.79) |
| 177.92 to <221.56 | 46 | 29 | 0.91 | (0.47, 1.73) |
| ≥221.56 | 46 | 79 | 2.47 | (1.38, 4.41) |
| *P trend^b^* |  |  |  | 0.0003 |
| **Free testosterone, nmol/L** | |  |  |  |
| <0.14 | 46 | 100 | Referent |  |
| 0.14 to <0.18 | 46 | 30 | 0.30 | (0.17, 0.53) |
| 0.18 to <0.22 | 45 | 24 | 0.25 | (0.13, 0.45) |
| ≥0.22 | 46 | 16 | 0.16 | (0.08, 0.31) |
| *P trend^b^* |  |  |  | <0.0001 |
| **Free DHT, pmol/L** |  |  |  |  |
| <11.99 | 45 | 110 | Referent |  |
| 11.99 to <16.35 | 46 | 33 | 0.29 | (0.17, 0.52) |
| 16.35 to <20.41 | 46 | 14 | 0.12 | (0.06, 0.25) |
| ≥20.41 | 46 | 13 | 0.12 | (0.06, 0.23) |
| *P trend^b^* |  |  |  | <0.0001 |
| **Free estradiol, pmol/L** |  |  |  |  |
| <1.27 | 46 | 116 | Referent |  |
| 1.27 to <1.51 | 46 | 22 | 0.19 | (0.10, 0.35) |
| 1.51 to <1.85 | 46 | 16 | 0.14 | (0.07, 0.27) |
| ≥1.85 | 46 | 16 | 0.14 | (0.07, 0.27) |
| *P trend^b^* |  |  |  | <0.0001 |

^a^Tests of linear trend were calculated by assigning the median of each quartile as scores. ^b^Progesterone values below the LOD form the referent with the subsequent three categories based on tertiles of the observed population distribution. ^c^Test of linear trend for progesterone was calculated by assigning the categorical groups as scores.
