# Supplementary material for: Association between circulating levels of sex steroid hormones and esophageal adenocarcinoma in the FINBAR Study
Source: PLoS One. 2018 Jan 17;13(1):e0190325. doi: 10.1371/journal.pone.0190325 (PMC5771564; doi:10.1371/journal.pone.0190325)
Supplement: S3 Table — aLogistic regression models were adjusted for age at interview (quartiles), education (<10, 10–12, 13–20 years), smoking (ever/never), BMI at interview (<25, 25–<30, ≥30 kg/m2), gastroesophageal reflux disease symptoms (yes/no), and H. pylori seropositivity (yes/no). bTests of linear trend were calculated by assigning the median of each quartile as scores. cProgesterone values below the LOD form the referent with the subsequent three categories based on tertiles of the observed population distribution. dTest of linear trend for progesterone was calculated by assigning the categorical groups as scores. (DOCX) [file pone.0190325.s003.docx]

**S3** **Table.** Adjusted^a^ odds ratios (ORs) and 95% confidence intervals (CI) for associations between circulating sex steroid hormone concentrations and esophageal adenocarcinoma risk, stratified by body mass index (kg/m^2^) change in the 5 years preceding interview.

|  |  |  | **EA cases, <-3.8 kg/m^2^** | | |  | **EA cases, -3.8 to <0 kg/m^2^** | | |  | **EA cases, ≥0 kg/m^2^** | | |
| --- | --- | --- | --- | --- | --- | --- | --- | --- | --- | --- | --- | --- | --- |
| **Hormone** | **Control (n)** |  | **Case (n)** | **OR** | **95% CI** |  | **Case (n)** | **OR** | **95% CI** |  | **Case (n)** | **OR** | **95% CI** |
| **DHEA, nmol/L** |  |  |  |  |  |  |  |  |  |  |  |  |  |
| <5.13 | 83 |  | 41 | Referent |  |  | 45 | Referent |  |  | 24 | Referent |  |
| ≥5.13 | 89 |  | 13 | 0.25 | (0.11, 0.57) |  | 19 | 0.45 | (0.21, 0.94) |  | 13 | 0.58 | (0.23, 1.50) |
| *P trend^1^* |  |  |  |  | 0.0005 |  |  |  | 0.3 |  |  |  | 0.7 |
| **Androstenediol, pmol/L** |  |  |  |  |  |  |  |  |  |  |  |  |  |
| <1764.39 | 83 |  | 37 | Referent |  |  | 48 | Referent |  |  | 28 | Referent |  |
| ≥1764.39 | 87 |  | 15 | 0.30 | (0.14, 0.65) |  | 16 | 0.29 | (0.14, 0.61) |  | 8 | 0.44 | (0.16, 1.19) |
| *P trend^1^* |  |  |  |  | <0.0001 |  |  |  | <0.0001 |  |  |  | 0.1 |
| **Androstenedione, nmol/L** | |  |  |  |  |  |  |  |  |  |  |  |  |
| <2.72 | 87 |  | 34 | Referent |  |  | 41 | Referent |  |  | 21 | Referent |  |
| ≥2.72 | 85 |  | 21 | 0.51 | (0.26, 1.03) |  | 24 | 0.51 | (0.26, 0.99) |  | 16 | 1.28 | (0.52, 3.10) |
| *P trend^1^* |  |  |  |  | 0.04 |  |  |  | 0.1 |  |  |  | 0.6 |
| **Testosterone, nmol/L** |  |  |  |  |  |  |  |  |  |  |  |  |  |
| <12.07 | 87 |  | 35 | Referent |  |  | 37 | Referent |  |  | 22 | Referent |  |
| ≥12.07 | 84 |  | 20 | 0.39 | (0.19, 0.79) |  | 27 | 0.59 | (0.30, 1.15) |  | 15 | 1.37 | (0.55, 3.43) |
| *P trend^1^* |  |  |  |  | 0.009 |  |  |  | 0.04 |  |  |  | 0.4 |
| **DHT, pmol/L** |  |  |  |  |  |  |  |  |  |  |  |  |  |
| <1008.55 | 85 |  | 39 | Referent |  |  | 46 | Referent |  |  | 24 | Referent |  |
| ≥1008.55 | 86 |  | 16 | 0.23 | (0.10, 0.49) |  | 19 | 0.26 | (0.13, 0.54) |  | 13 | 0.85 | (0.34, 2.15) |
| *P trend^1^* |  |  |  |  | <0.0001 |  |  |  | 0.0002 |  |  |  | 0.6 |
| **ADT, pmol/L** |  |  |  |  |  |  |  |  |  |  |  |  |  |
| <592.55 | 81 |  | 35 | Referent |  |  | 43 | Referent |  |  | 17 | Referent |  |
| **≥**592.55 | 83 |  | 11 | 0.30 | (0.13, 0.67) |  | 12 | 0.30 | (0.14, 0.64) |  | 13 | 0.91 | (0.36, 2.29) |
| *P trend^1^* |  |  |  |  | 0.0005 |  |  |  | 0.002 |  |  |  | 0.6 |
| **Estrone, pmol/L** |  |  |  |  |  |  |  |  |  |  |  |  |  |
| <104.23 | 86 |  | 41 | Referent |  |  | 46 | Referent |  |  | 16 | Referent |  |
| ≥104.23 | 86 |  | 13 | 0.36 | (0.17, 0.76) |  | 15 | 0.32 | (0.15, 0.65) |  | 20 | 1.32 | (0.55, 3.12) |
| *P trend^1^* |  |  |  |  | 0.003 |  |  |  | 0.001 |  |  |  | 0.5 |
| **Estradiol, pmol/L** |  |  |  |  |  |  |  |  |  |  |  |  |  |
| <67.33 | 88 |  | 43 | Referent |  |  | 51 | Referent |  |  | 24 | Referent |  |
| ≥67.33 | 84 |  | 12 | 0.32 | (0.15, 0.68) |  | 13 | 0.27 | (0.13, 0.57) |  | 13 | 0.57 | (0.24, 1.39) |
| *P trend^1^* |  |  |  |  | 0.0007 |  |  |  | <0.0001 |  |  |  | 0.5 |
| **Progesterone^2^, nmol/L** |  |  |  |  |  |  |  |  |  |  |  |  |  |
| <0.20 | 115 |  | 32 | Referent |  |  | 32 | Referent |  |  | 31 | Referent |  |
| ≥0.20 | 57 |  | 23 | 1.37 | (0.69, 2.72) |  | 33 | 2.18 | (1.13, 4.18) |  | 7 | 0.55 | (0.21, 1.45) |
| *P trend^1^* |  |  |  |  | 0.8 |  |  |  | 0.1 |  |  |  | 0.1 |
| **SHBG, nmol/L** |  |  |  |  |  |  |  |  |  |  |  |  |  |
| <53.70 | 85 |  | 13 | Referent |  |  | 24 | Referent |  |  | 18 | Referent |  |
| ≥53.70 | 86 |  | 42 | 2.36 | (1.10, 5.05) |  | 41 | 1.27 | (0.65, 2.49) |  | 20 | 0.95 | (0.40, 2.25) |
| *P trend^1^* |  |  |  |  | 0.002 |  |  |  | 0.1 |  |  |  | 0.5 |
| **Parent estrogens, pmol/L** | |  |  |  |  |  |  |  |  |  |  |  |  |
| <175.22 | 86 |  | 43 | Referent |  |  | 47 | Referent |  |  | 18 | Referent |  |
| ≥175.22 | 86 |  | 11 | 0.26 | (0.12, 0.57) |  | 14 | 0.28 | (0.13, 0.58) |  | 18 | 0.90 | (0.38, 2.13) |
| *P trend^1^* |  |  |  |  | 0.0002 |  |  |  | <0.0001 |  |  |  | 0.7 |
| **Testosterone: Parent estrogens ratio** | | | |  |  |  |  |  |  |  |  |  |  |
| <68.78 | 84 |  | 23 | Referent |  |  | 18 | Referent |  |  | 17 | Referent |  |
| ≥68.78 | 87 |  | 31 | 0.83 | (0.40, 1.71) |  | 43 | 2.30 | (1.12, 4.73) |  | 19 | 1.98 | (0.76, 5.16) |
| *P trend^1^* |  |  |  |  | 0.6 |  |  |  | 0.004 |  |  |  | 0.2 |
| **Androstenedione: Estrone ratio** | |  |  |  |  |  |  |  |  |  |  |  |  |
| <24.49 | 84 |  | 20 | Referent |  |  | 18 | Referent |  |  | 21 | Referent |  |
| ≥24.49 | 88 |  | 34 | 1.15 | (0.56, 2.40) |  | 43 | 2.40 | (1.15, 5.01) |  | 15 | 1.10 | (0.44, 2.75) |
| *P trend^1^* |  |  |  |  | 0.6 |  |  |  | 0.01 |  |  |  | 0.9 |
| **Testosterone: Estradiol ratio** | |  |  |  |  |  |  |  |  |  |  |  |  |
| <177.92 | 84 |  | 20 | Referent |  |  | 16 | Referent |  |  | 20 | Referent |  |
| ≥177.92 | 87 |  | 35 | 1.07 | (0.52, 2.20) |  | 48 | 2.61 | (1.27, 5.38) |  | 17 | 1.35 | (0.54, 3.39) |
| *P trend^1^* |  |  |  |  | 0.4 |  |  |  | 0.0005 |  |  |  | 0.3 |
| **Free testosterone, nmol/L** | |  |  |  |  |  |  |  |  |  |  |  |  |
| <0.18 | 86 |  | 44 | Referent |  |  | 54 | Referent |  |  | 21 | Referent |  |
| ≥0.18 | 84 |  | 11 | 0.22 | (0.10, 0.50) |  | 10 | 0.18 | (0.08, 0.40) |  | 16 | 1.37 | (0.54, 3.46) |
| *P trend^1^* |  |  |  |  | <0.0001 |  |  |  | <0.0001 |  |  |  | 0.7 |
| **Free DHT, pmol/L** |  |  |  |  |  |  |  |  |  |  |  |  |  |
| <16.35 | 83 |  | 51 | Referent |  |  | 55 | Referent |  |  | 27 | Referent |  |
| ≥16.35 | 87 |  | 4 | 0.05 | (0.02, 0.16) |  | 9 | 0.12 | (0.05, 0.29) |  | 10 | 0.43 | (0.17, 1.11) |
| *P trend^1^* |  |  |  |  | <0.0001 |  |  |  | <0.0001 |  |  |  | 0.1 |
| **Free estradiol, pmol/L** |  |  |  |  |  |  |  |  |  |  |  |  |  |
| <1.51 | 85 |  | 46 | Referent |  |  | 58 | Referent |  |  | 23 | Referent |  |
| ≥1.51 | 86 |  | 9 | 0.26 | (0.11, 0.60) |  | 6 | 0.12 | (0.05, 0.32) |  | 14 | 0.61 | (0.25, 1.53) |
| *P trend^1^* |  |  |  |  | <0.0001 |  |  |  | <0.0001 |  |  |  | 0.2 |

^a^Logistic regression models were adjusted for age at interview (quartiles), education (<10, 10–12, 13–20 years), smoking (ever/never), BMI at interview (<25, 25–<30, ≥30 kg/m2), gastroesophageal reflux disease symptoms (yes/no), and *H. pylori* seropositivity (yes/no). ^b^Tests of linear trend were calculated by assigning the median of each quartile as scores. ^c^Progesterone values below the LOD form the referent with the subsequent three categories based on tertiles of the observed population distribution. ^d^Test of linear trend for progesterone was calculated by assigning the categorical groups as scores.
