# Supplementary material for: Association between circulating levels of sex steroid hormones and esophageal adenocarcinoma in the FINBAR Study
Source: PLoS One. 2018 Jan 17;13(1):e0190325. doi: 10.1371/journal.pone.0190325 (PMC5771564; doi:10.1371/journal.pone.0190325)
Supplement: S4 Table — aLogistic regression models were adjusted for age at interview (continuous), education (<10, 10–12, 13–20 years), smoking (ever/never), BMI at interview (<25, 25–<30, ≥30 kg/m2), gastroesophageal reflux disease symptoms (yes/no), and H. pylori seropositivity (yes/no). bStandardized to half the value of the interquartile range (e.g., for DHEA the OR is 0.70 for an increase of 2.41 nmol/L, which is 0.5*[8.18−3.36]), which approximates a single quartile increase in exposure. (DOCX) [file pone.0190325.s004.docx]

| **S4 Table.** Adjusted^a^ odds ratios (ORs) and 95% confidence intervals (CIs) for the associations between circulating metabolite concentrations^b^ and esophageal adenocarcinoma incidence, Factors Influencing the Barrett/Adenocarcinoma Relationship: 2002–2004. | | | | | |
| --- | --- | --- | --- | --- | --- |
| **Hormone** | **Control (n)** | **Esophageal Adeno. (n)** | **OR** | **95% CI** | **P value** |
| **DHEA, nmol/L** | 172 | 155 | 0.70 | (0.56, 0.87) | 0.001 |
| **Androstenediol, pmol/L** | 170 | 152 | 0.61 | (0.50, 0.75) | <0.0001 |
| **Androstenedione, nmol/L** | 172 | 157 | 0.96 | (0.85, 1.08) | 0.5 |
| **Testosterone, nmol/L** | 171 | 156 | 0.92 | (0.81, 1.04) | 0.2 |
| **DHT, pmol/L** | 171 | 157 | 0.77 | (0.66, 0.91) | 0.002 |
| **ADT, pmol/L** | 164 | 131 | 0.71 | (0.58, 0.86) | 0.0006 |
| **Estrone, pmol/L** | 172 | 151 | 0.79 | (0.67, 0.93) | 0.006 |
| **Estradiol, pmol/L** | 172 | 156 | 0.72 | (0.61, 0.84) | <0.0001 |
| **SHBG, nmol/L** | 171 | 158 | 1.26 | (1.12, 1.42) | 0.0002 |
| **Parent estrogens, pmol/L** | 172 | 151 | 0.73 | (0.62, 0.87) | 0.0003 |
| **Testosterone: Parent estrogens ratio** | 171 | 151 | 1.16 | (1.02, 1.32) | 0.02 |
| **Androstenedione: Estrone ratio** | 172 | 151 | 1.12 | (1.01, 1.25) | 0.03 |
| **Testosterone: Estradiol ratio** | 171 | 156 | 1.25 | (1.09, 1.43) | 0.002 |
| **Free testosterone, nmol/L** | 170 | 156 | 0.67 | (0.56, 0.80) | <0.0001 |
| **Free DHT, pmol/L** | 170 | 156 | 0.57 | (0.47, 0.69) | <0.0001 |
| **Free estradiol, pmol/L** | 171 | 156 | 0.59 | (0.50, 0.71) | <0.0001 |
| ^a^Logistic regression models were adjusted for age at interview (continuous), education (<10, 10–12, 13–20 years), smoking (ever/never), BMI at interview (<25, 25–<30, ≥30 kg/m2), gastroesophageal reflux disease symptoms (yes/no), and *H. pylori* seropositivity (yes/no). ^b^Standardized to half the value of the interquartile range (e.g., for DHEA the OR is 0.70 for an increase of 2.41 nmol/L, which is 0.5*[8.18−3.36]), which approximates a single quartile increase in exposure. | | | | | |
